# Introduction to the Special Issue on Adverse Childhood Experiences (ACEs): Prevention, Intervention, and Access to Care

**DOI:** 10.3390/children9081205

**Published:** 2022-08-11

**Authors:** Kimberly Burkhart, Carolyn E. Ievers-Landis, Alissa Huth-Bocks

**Affiliations:** 1 Department of Pediatrics, UH Rainbow Babies & Children’s Hospital, Cleveland, OH 44106, USA; 2 School of Medicine, Case Western Reserve University, Cleveland, OH 44106, USA

Exposure to adverse childhood experiences (ACEs) is an international public health problem. Approximately two-thirds of all children experience at least one form of adversity prior to 18 years of age. It has become widely accepted over the last 20 years that childhood exposure to traumatic events affects cognitive, affective, relational, and behavioral development. These traumatic experiences have been categorized as exposure to child abuse and/or neglect; household challenges or dysfunction, including a range of chronic stressors such as parental absence, family mental illness, and parental incarceration; and other severe stressors such as poverty and community violence. Exposure to such stressors, especially those that are prolonged during formative years of development, may lead to altered activation of the glucocorticoid stress response, increased cortisol production, and disruption of the neuroendocrine and immune systems, which can lead to changes in neurological pathways in the hippocampus, amygdala, and prefrontal cortex. These biological and physiological changes (often referred to as ‘toxic stress’) can then manifest as long-term mental and physical health problems (e.g., anxiety, depression, posttraumatic stress disorder, substance use, risky behavior, obesity, cancer, and suicide attempts).

The articles selected for this Special Issue of *Children* highlight the intergenerational nature of ACEs; the types and sequelae of ACEs in special populations of children, including those with neurodevelopmental disorders; and the importance of trauma-informed healthcare settings for prevention and early identification of ACEs. The intent of this editorial is to synthesize the results of the collection of articles in this Special Issue and to propose areas for further investigation.

The experience of ACEs in childhood increases the likelihood of adversity for one’s own children, contributing to a legacy of trauma within families. This cross-generational transmission of trauma is most commonly associated with parental history of maltreatment in childhood and problematic parenting behavior in adulthood. In this Special Issue, Howell et al. [1] reviewed research in this important area. Studies suggest that pregnancy is a particularly vulnerable time for both the mother and fetus to be exposed to ACEs, as there is an association between adverse exposures and low birth weight, premature delivery, poor child cognitive and socioemotional development, as well as early difficulties in the parent–child relationship. Research by Schickedanz and colleagues [2] also addressed intergenerational transmission by examining the association between a national sample of parents’ ACE scores and their adult children’s ACE scores. The results indicated that, when adjusting for demographic factors and socioeconomic status, parents’ ACE counts positively correlated with their children’s ACE counts. Partial mediators of the intergenerational transmission of ACEs included poor parental mental health, aggravation toward their children and with parenting (i.e., stress experienced by parents associated with caring for their children), and parenting conflict. In Howell et al.’s [1] article, the authors discussed research findings related to how community factors such as racial discrimination, social inequity, historical trauma, and community violence may exacerbate the potentially detrimental effects of intergenerational trauma. Thus, these two papers go beyond identifying intergenerational transmission to highlighting important mediators and moderators to examine further. 

The articles by Zarei et al. [3] and Ronis et al. [4] both elucidated the relationship between ACEs, and neurodevelopmental and behavioral conditions in children using the National Survey of Children’s Health (NSCH). Specifically, Zarei and colleagues [3] identified dose-dependent associations between ACEs and multiple neurodevelopmental and behavioral conditions such as behavior problems, depression, and autism spectrum disorder among 0–5-year-old children. Although their study was a cross-sectional study, the use of a cumulative risk methodology highlights the vital need for early screening and identification of children who might be at the most high risk for behavioral health conditions so that effective treatments can commence as soon as possible. Moreover, in this study, US children with ASD were found to be twice as likely as their neurotypical peers to have two or more social adversities and three times more likely to report two or more relational adversities. The findings from Ronis et al. [4] suggest that even one adverse experience may be enough to influence treatment and type of healthcare utilization among 0–17-year-old children, as reported by caregivers. Interestingly, children with ASD who were taking medications to treat their symptoms had a significantly higher likelihood of having experienced relational adversities (most often that the parents were divorced or were frequently angry/aggravated with the child) than those who were not taking medications. This finding highlights the importance of exploring whether the severity of ASD symptoms in children who were on medications was worse, if the parents’ ability to tolerate their children’s symptoms was compromised, or if other factors were at play. Regardless, both studies highlight the importance of screening for ACEs from an early age, particularly for these high-risk groups.

It is clear that ACEs are common and can pose risks to health and development. Current research, including the articles in this Special Issue, have begun to examine more complex questions such as the transmission of ACEs, their mechanisms of effects, and their manifestation in special populations. As awareness has grown, so has a ‘call’ to put policies and practices for trauma-informed care in place. In 2012, the American Academy of Pediatrics issued a recommendation for taking a two-generation approach to identifying high-risk families that involves assessing children and their parents about experiences with early adversity. Despite this recommendation, only 15% of pediatric practices regularly screen for more than two-parent ACEs. Screening for ACEs has been somewhat controversial, with some noted concerns regarding costs and potential negative effects of screening (e.g., lack of training for proper screening and lack of resources when adversity is identified). It is the position of the guest editors of this Special Issue that screening is a necessary and helpful component to a more comprehensive trauma-informed healthcare system. Four papers (Racine et al. [5], Herrero-Roldan et al. [6], Olecka [7], and Matthew et al. [8]) in this issue were devoted to identifying the benefits of preventing and implementing a trauma-informed approach to care.

A trauma-informed care (TIC) model consists of many components, including screening for adverse events, training of staff to recognize the impact of trauma on health, and employing strategies to avoid re-victimization and to promote healing. Limited research exists on TIC approaches implemented within the prenatal care setting. Studies such as Racine et al. [5] have identified some associations between TIC approaches and positive health aspects in infant outcomes. Specifically, as reported in Racine et al. [5], comparing groups receiving standard care versus a TIC approach at two points in time, infants of mothers receiving TIC had significantly fewer adverse birth outcomes. Whether the TIC approach was a direct contributor to these improved outcomes is unknown, but this finding warrants additional investigation. Other findings show that a TIC approach for pregnant women in primary care is related to increased attendance at prenatal appointments and decreased rates of preterm birth compared with rates prior to the implementation of the TIC approach. Moreover, in addition to traditional ACEs, Olecka’s study [7] identified parental risk factors, including poor parenting skills, mental disorder or cognitive deficit on the part of the parent, addiction presence, and the lack of an emotional bond, as being risk factors for violence and fatal child abuse for children younger than 5 years old. Based on these results, prevention efforts should include particular attention to children not registered with a pediatrician and to those who fail to attend regular medical examinations. Early diagnosis of child neglect and abuse is an ongoing challenge. As such, Herrero-Roldan and colleagues [6] suggested that a reasonable approach to prevention and detection is to assess for risk and protective factors as part of a review of family demographics, such as the parent’s life adversity, parental mental health, and quality of mother–child availability via screening in primary care. Identifying a potentially modifiable protective factor, in this case dyadic synchrony, is a notable contribution of this study, as this early sign of relational health could be included in early intervention strategies.

Based on qualitative data obtained in Matthew and colleagues’ study [8], there are both facilitating factors and logistical barriers to implementing trauma-informed care. Continuity among providers and approaches, additional time allotted for adversity screening, proper training, and access to psychosocial resources are perceived as being critical by both providers/staff and patients. Staff support and resources are necessary for feasible delivery of TIC including patient access to resources to address basic needs and psychosocial services, and opportunities for reflective supervision for staff.

Screening children and their parents for ACEs holds the potential for interrupting the intergenerational cycle of adversity and the downstream negative physical and mental health consequences of exposure. Universal parenting prevention programs (e.g., Healthy Steps and Triple P) could serve to minimize the risk of child maltreatment. Moreover, the findings of this Special Issue support two-generation approaches in identifying, addressing, and treating the physical and psychological consequences of childhood adversities. It is recommended that screening expands beyond the original ACEs identified by Felitti and colleagues, i.e., child maltreatment (caregiver physical, sexual, and psychological abuse, and physical and emotional neglect) and household dysfunction (caregiver substance use, mental illness, incarceration, divorce, and exposure to familial intimate partner violence), to reflect community and societal adversities. Options for comprehensive screening include the Structured Problem Analysis of Raising Kids (SPARK) and a Safe Environment for Every Kid (SEEK). It is proposed by the authors of this editorial that the broader social ecology of children’s experience impact physical health and psychological well-being. Screening should include exposure to community and macro-level adversity including financial hardship, bullying, community violence, discrimination, racism, consequences of war and conflict, and both natural and manmade disasters.

Future studies should examine protective factors such as parenting support, positive school climates, close-knit communities, and safe neighborhoods as buffers against the transmission of intergenerational ACEs. Further exploration could include the strength of specific adverse and resilience factors as predictors of child psychological and physical well-being. Additional outcomes such as parenting stress, perceived social support, and child development should be investigated when receiving care in a trauma-informed two-generation medical model. Further work should expand on the examination of adversity-exposed youth with neurodevelopmental disorders and identification of the predisposing and enabling factors that influence access to care and healthcare utilization.

In summary, a multilevel and intergenerational approach would optimally guide efforts surrounding ACEs. Biological, psychological, and sociological perspectives would improve the understanding of the negative impact of adversity and inform the development of community-based approaches for prevention and intervention. This Special Issue offers further support that a feasible, acceptable, and helpful model for both healthcare providers and patients is screening and preventing adversity through the implementation of a trauma-informed two-generation medical model.
